# End stage renal disease is associated with development of dementia

**DOI:** 10.18632/oncotarget.22458

**Published:** 2017-11-15

**Authors:** Peir-Haur Hung, Chih-Ching Yeh, Chih-Yen Hsiao, Pi-Shan Sung, Chih-Hsin Muo, Fung-Chang Sung, Kuan-Yu Hung, Kuen-Jer Tsai

**Affiliations:** ^1^ Department of Internal Medicine, Ditmanson Medical Foundation Chia-yi Christian Hospital, Chia-yi, Taiwan; ^2^ Department of Applied Life Science and Health, Chia-Nan University of Pharmacy and Science, Tainan, Taiwan; ^3^ School of Public Health, College of Public Health and Nutrition, Taipei Medical University, Taipei, Taiwan; ^4^ Department of Public Health, China Medical University, Taichung, Taiwan; ^5^ Department of Hospital and Health Care Administration, Chia-Nan University of Pharmacy and Science, Tainan, Taiwan; ^6^ Institute of Clinical Medicine, College of Medicine, National Cheng Kung University, Tainan, Taiwan; ^7^ Department of Neurology, National Cheng Kung University Hospital, Tainan, Taiwan; ^8^ Management Office for Health Data, China Medical University Hospital, Taichung, Taiwan; ^9^ Graduate Institute of Clinical Medical Science, School of Medicine, College of Medicine, China Medical University, Taichung, Taiwan; ^10^ Department of Internal Medicine, National Taiwan University Hospital, Hsin-Chu Branch, Hsin-Chu City, Taiwan; ^11^ Center of Clinical Medicine, National Cheng Kung University Hospital, College of Medicine, National Cheng Kung University, Tainan, Taiwan

**Keywords:** Alzheimer’s disease, end stage renal disease, hemodialysis, peritoneal dialysis, dementia, Gerotarget

## Abstract

There is controversy regarding the extent of risk for dementia in patients with end stage renal disease (ESRD) who are undergoing hemodialysis (HD) or peritoneal dialysis (PD). We examined data from Taiwan's Longitudinal Health Insurance Database, and used Cox proportional hazard regression analysis to compare the development of dementia in 72,934 HD and PD patients with 72,934 matched controls from January 1, 1999 to December 31, 2010. The results indicate an increased risk for dementia overall in HD patients (adjusted hazard ratio [aHR] = 1.64, *p* < 0.0001) and PD patients (aHR = 2.21, *p* < 0.0001). HD and PD patients also had significantly greater aHRs for vascular dementia (VaD) and unspecified dementia (UnD), but only HD patients had a significantly greater aHR for Alzheimer's disease (AD). Further research is needed to confirm whether management of ESRD with PD can reduce the incidence of AD, and to identify the mechanisms by which ESRD increases the risk of dementia.

## INTRODUCTION

Dementia is a major cause of death and disability among elderly individuals in the general population [1], and advanced age, cerebrovascular disease, and cardiovascular risk factors (diabetes, hypertension, hyperlipidemia, and smoking) increase the risk for dementia [2]. Recent studies suggest that individuals with end-stage renal disease (ESRD) have a 2–7-fold higher prevalence of cognitive impairment and dementia than the general population [3–5].

The pathogenic mechanisms responsible for the relationship between cognitive decline and kidney dysfunction may be similar to those responsible for relationship between cognitive decline and other cardiovascular disorders, such as atherosclerosis, clinical stroke, silent stroke, oxidative stress, and white matter lesions [6–8]. Other pathological conditions, such as neurotoxicity of the uremic state, may also explain the relationship of chronic kidney disease (CKD) with cognitive disorders such as Alzheimer's disease (AD), especially in the absence of obvious cerebrovascular disease [9]. Previous studies of patients with ESRD have determined that several uremia-related factors increase the risk for cognitive impairment, including insufficient dialysis, anemia, and aluminum exposure [10–12], although not all of these findings have been confirmed.

The general objective of our research was to determine whether the association of ESRD with cognitive decline can be explained by medical comorbidities or mode of dialysis. Thus, we used the Longitudinal Health Insurance Database (LHIRD) of Taiwan to determine the risk of dementia in ESRD patients undergoing hemodialysis (HD) or peritoneal dialysis (PD) relative to matched healthy controls, and we also analyzed the relationships of HD and PD with 3 different dementia subtypes.

## RESULTS

After propensity score matching, we identified 72,934 ESRD patients (63,372 undergoing HD and 9,562 undergoing PD) and 72,934 control subjects. Table 1 shows the demographic characteristics and comorbidities of these subjects. The only significant difference was that the HD and PD patients had shorter mean follow-up times than the control subjects (standardized mean difference (SMD) = 0.427 and SMD = 0.705, respectively).

**Table 1:** Demographic characteristics and comorbidities of the ESRD and control groups

	HD				Standardized difference	PD				Standardized difference
	Yes (*N* = 63372)		No (*N* = 63372)			Yes (*N* = 9562)		No (*N* = 9562)		
	*n*	%, (SD)	*n*	%, (SD)		*n*	%, (SD)	*n*	%, (SD)	
Mean age, years (SD)	61.2	(13.9)	62.2	(13.7)	0.069	52.9	(15.0)	54.0	(14.3)	0.072
Sex										
Male	32389	48.9	31788	50.2	0.019	4435	46.4	4414	46.2	0.004
Female	30983	51.1	31584	49.8	0.019	5127	53.6	5148	53.8	0.004
Residence										
Urban	34492	54.4	34362	54.2	0.04	5988	62.6	5918	61.9	0.015
Suburban	20927	33.0	20895	33.0	0.01	2760	28.9	2844	29.7	0.019
Rural	7953	12.6	8115	12.8	0.01	814	8.51	800	8.37	0.019
Mean follow-up, years (SD)	4.24	(3.15)	5.65	(3.45)	**0.427**	3.23	(2.43)	5.25	(3.24)	**0.705**
Coronary heart disease	24360	38.4	23400	36.9	0.031	2804	29.3	2939	30.7	0.031
Hypertension	56250	88.8	56422	89.0	0.009	8507	89.0	8521	89.1	0.005
Diabetes	28271	44.6	28584	45.1	0.010	3570	37.3	3720	38.9	0.032
Atrial fibrillation	1128	1.78	1434	2.26	0.034	111	1.16	96	1.00	0.015
Heart failure	7744	12.2	7077	11.2	0.034	1291	13.5	1431	15.0	0.015
Hyperlipidemia	26851	42.4	27620	43.6	0.025	4231	44.3	4303	45.0	0.015
Mean propensity score (SD)	0.37	(0.22)	0.37	(0.22)	0.001	0.11	(0.10)	0.11	(0.10)	0.001

The HD and PD groups were each age- and sex-matched with controls who did not have ESRD

Table 2 shows the crude hazard ratios (HRs) and adjusted HRs (aHRs) for dementia in the different groups during the study period. In the entire study population of 145,868 patients, 9751 patients (6.68%) developed dementia, 5009 (6.87%) in the ESRD cohort and 4752 (6.52%) in the control cohort. A total of 4706 HD patients (7.43%) and 303 PD patients (3.17%) developed dementia. Cox proportional hazard regression analysis indicated the aHR for dementia was 1.64 (95% confidence interval (CI) = 1.58 to 1.71; *p* < 0.0001) in HD patients and 2.21 (95% CI = 1.87 to 2.62; *p* < 0.0001) in PD patients.

**Table 2:** Crude and adjusted hazard ratios for development of dementia over 12 years in ESRD patients undergoing HD or PD relative to matched controls

	Control group	HD group	*P*	Control group	PD group	*P*
No. of cases	4420	4706		322	303	
Person-year	358187	268868		50205	30916	
Incidence rate (10^−3^)	12.34	17.50		6.41	9.80	
Crude HR (95% CI)	1.00	1.40 (1.35–1.46)	**< 0.0001**	1.00	1.53 (1.30–1.80)	**< 0.0001**
Adjusted HR (95% CI)^a^	1.00	1.64 (1.58–1.71)	**< 0.0001**	1.00	2.21 (1.87–2.62)	**< 0.0001**

Adjustments were made for age, sex, residence, diabetes, hypertension, coronary heart disease, heart failure, atrial fibrillation, and hyperlipidemia.

**Figure 1:**
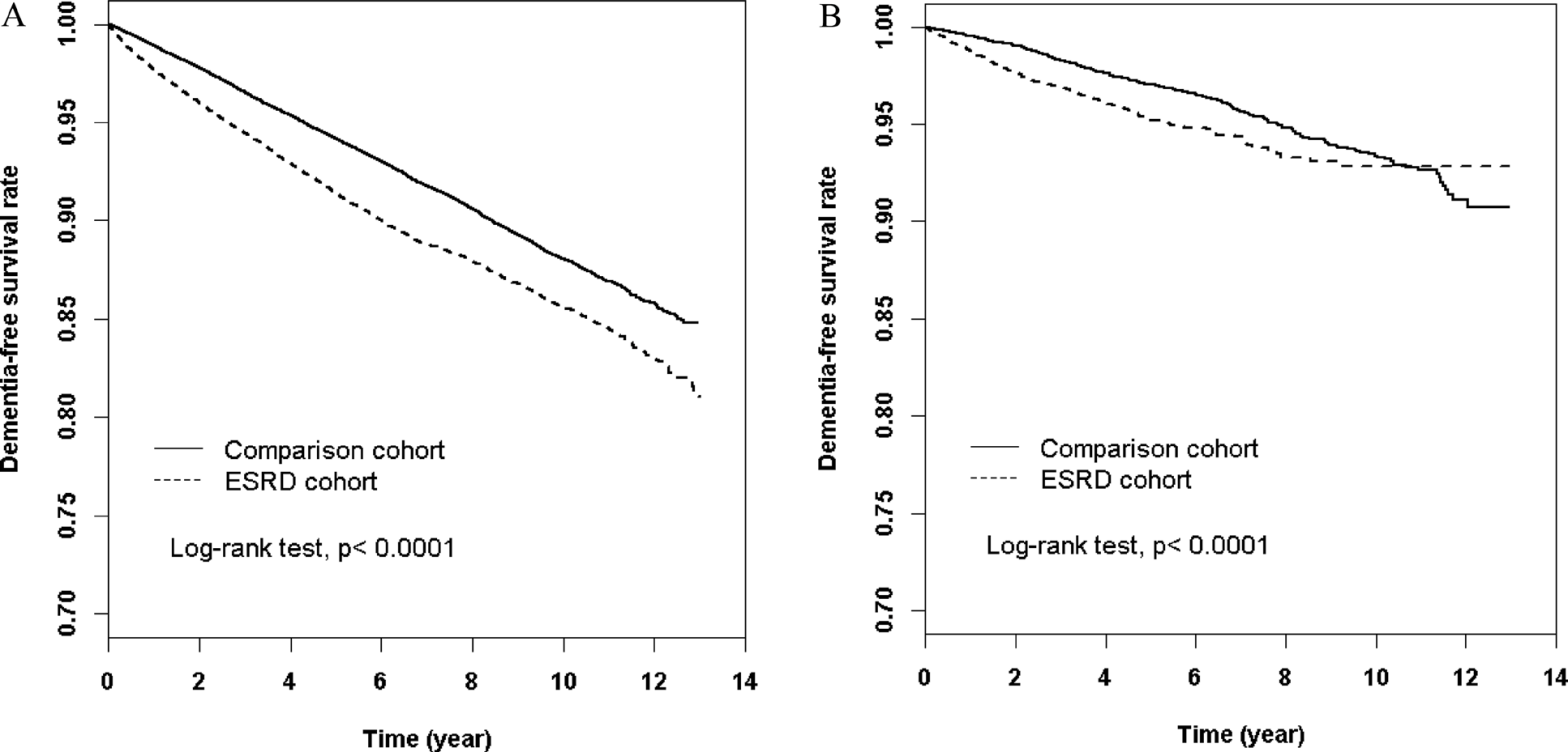
Dementia-free survival rate of ESRD patients Undergoing HD (**A**) and PD (**B**) relative to age-and sex-matched controls.

Figure 1 shows the dementia-free survival curves for the HD cohort (top) and PD cohort (bottom) relative to propensity score-matched controls. Both comparisons indicate that patients undergoing dialysis had significantly increased risk of dementia.

The multivariate Cox model shows that the risk of dementia increased with age in the HD and PD groups (Table 3). For HD patients, the risk of dementia was significantly greater for females (aHR = 1.10, 95% CI = 1.05 to 1.14), and for those with diabetes mellitus (aHR = 1.28, 95% CI = 1.23 to 1.34), hypertension (aHR = 1.23, 95% CI =1.13 to 1.34), coronary heart disease (CHD) (aHR = 1.20, 95% CI =1.15 to 1.26), or heart failure (HF) (aHR = 1.26, 95% CI =1.18 to 1.33). For PD patients, the risk of dementia was significantly greater for those with diabetes mellitus (aHR = 1.36, 95% CI = 1.15 to 1.61) and CHD (aHR = 1.30, 95% CI = 1.09 to 1.55).

**Table 3:** Multivariate Cox proportional hazards model for development of dementia over 12 years in ESRD patients undergoing HD or PD relative to matched controls

	HD group		PD group	
	aHR (95% CI)	*P*	aHR (95% CI)	*P*
Age, years				
18–44	1.00		1.00	
45–64	6.62 (5.29–8.29)	**< 0.0001**	7.02 (3.97–12.4)	**< 0.0001**
≥ 65	36.3 (29.1–45.3)	**< 0.0001**	57.8 (32.9–101)	**< 0.0001**
Sex				
Male	1.00		1.00	
Female	1.10 (1.05–1.14)	**< 0.0001**	1.13 (0.96–1.33)	0.14
Residence				
Urban	1.03 (0.98–1.07)	0.27	1.04 (0.87–1.24)	0.70
Suburban	1.00		1.00	
Rural	1.02 (0.96–1.09)	0.53	1.06 (0.80–1.41)	0.69
HD	1.64 (1.58–1.71)	**< 0.0001**	--	
PD	--		2.21 (1.87–2.62)	**< 0.0001**
Diabetes	1.28 (1.23–1.34)	**< 0.0001**	1.36 (1.15–1.61)	**0.0003**
Hypertension	1.23 (1.13–1.34)	**< 0.0001**	1.02 (0.72–1.43)	0.93
Coronary heart disease	1.20 (1.15–1.26)	**< 0.0001**	1.30 (1.09–1.55)	**0.003**
Atrial fibrillation	1.10 (0.97–1.24)	0.15	0.55 (0.27–1.12)	0.20
Heart failure	1.26 (1.18–1.33)	**< 0.0001**	1.17 (0.97–1.42)	0.11
Hyperlipidemia	0.98 (0.94–1.02)	0.37	1.12 (0.94–1.32)	0.20

Table 4 shows the crude HRs and aHRs for development of 3 different subtypes of dementia in the HD and PD cohorts. In comparison with the controls, HD patients were more likely to develop each subtype of dementia (AD: aHR = 1.30, *p* = 0.01; vascular dementia (VaD): aHR = 1.57, *p* < 0.0001; unspecified dementia (UnD): aHR = 1.67, *p* < 0.0001). However, PD patients were more likely to develop VaD (aHR = 2.08, *p* = 0.002) and UnD (aHR = 2.26, *p* < 0.0001), but not AD (aHR = 1.77, *p* = 0.14).

**Table 4:** Crude and adjusted hazard ratios for development of different dementia subtypes over 12 years in ESRD patients undergoing HD or PD relative to matched controls

	Control group	HD group	*P*	Control group	PD group	*P*
**Alzheimer's disease**						
No. of cases	202	168		19	13	
Crude HR (95% CI)	1.00	1.11 (0.90–1.36)	0.34	1.00	1.23 (0.60–2.53)	0.58
Adjusted HR (95% CI)^a^	1.00	1.30 (1.06–1.60)	**0.01**	1.00	1.77 (0.83–3.80)	0.14
**Vascular dementia**						
No. of cases	559	567		48	41	
Crude HR (95% CI)	1.00	1.33 (1.18–1.50)	**< 0.0001**	1.00	1.40 (0.92–2.15)	0.12
Adjusted HR (95% CI)^a^	1.00	1.57 (1.39–1.77)	**< 0.0001**	1.00	2.08 (1.32–3.28)	**0.002**
**Unspecified dementia**						
No. of cases	3659	3971		255	249	
Crude HR (95% CI)	1.00	1.43 (1.37–1.50)	**< 0.0001**	1.00	1.58 (1.32–1.88)	**< 0.0001**
Adjusted HR (95% CI)^a^	1.00	1.67 (1.60–1.75)	**< 0.0001**	1.00	2.26 (1.87–2.73)	**< 0.0001**

Adjustments were made for significant variables from Table 1 (patient age, gender, urbanization level, diabetes, hypertension, coronary heart disease, heart failure, atrial fibrillation, and hyperlipidemia).

## DISCUSSION

To our knowledge, the present study is the first nationwide population-based cohort study to compare the incidence rates of AD, VaD, and UnD in patients undergoing HD and PD with matched controls from the general population. More specifically, we used multivariable regression analysis to retrospectively compare the development of dementia over a 12 year period in ESRD patients undergoing HD or PD with controls who did not have ESRD. The results indicate that ESRD patients undergoing HD or PD have a significantly increased risk for dementia. Moreover, our analysis of dementia subtypes indicated that HD and PD patients have increased risk for VaD and UnD, and that HD patients- but not PD patients- have an increased risk for AD.

Our results clearly indicate an increased risk of dementia in the HD and PD cohorts relative to the age- and sex-matched controls. It is possible that ESRD and dementia share several pathological pathways, and develop simultaneously during aging due to the accumulation of “traditional” vascular risk factors. Alternatively, ESRD may lead to dementia due to “nontraditional” vascular risk factors caused by renal impairment, such as anemia, oxidative stress, chronic inflammation, and uremic toxins [13]. These nontraditional factors are associated with generalized endothelial dysfunction and systemic vascular remodeling [14, 15]. Moreover, dementia and ESRD may also be similar in that they have certain genetic factors in common and in that each is characterized by the accumulation of certain metabolic toxins [16]. Indeed, a recent meta-analysis of 10 prospective studies showed a significant association of CKD with cognitive impairment [17]. Furthermore, vascular injury and damage from a stroke or white-matter lesion contributes greatly to cognitive decline in AD [18]. Therefore, the findings of the present study provide a basis for investigating the underlying neuropathophysiological mechanisms that link ESRD with the development of dementia.

HD and PD each has its advantages and disadvantages, in that each has different impacts on a patient's physical, psychological, and social health, and each places unique limitations on a patient's lifestyle [19]. The limited available data suggest that the prevalence of cognitive impairment might be lower in PD patients than in HD patients [20, 21]. Other studies showed that patients with CKD had a higher prevalence of silent cerebral infarctions and a greater degree of brain atrophy [22], and that these changes were apparently related to the duration of HD [23]. In addition, hemodynamic changes related to HD, including a decline in blood pressure, acute metabolic and fluid changes, and more efficient HD (indicated by Kt/V), are associated with a higher incidence of cognitive dysfunction [24]. However, a previous direct comparison of HD and PD patients indicated no significant difference in the development of ischemic stroke [25], possibly explaining why more dialysis patients experience VaD than AD [3], as well as our finding that HD and PD patients had a significantly increased risk of VaD and UnD (Table 4).

Our results indicate that PD and HD patients had greater risk for VaD and UnD than controls, and that the aHRs for both of these subtypes of dementia were greater for PD patients than HD patients. HD and PD generally differ in efficiency (*i.e.* removal of uremic toxins) and have different effects on patients’ physical, psychological, and social health. Although a rapid fluid and electrolyte shift occurs during HD but not PD, PD can lead to fluid overloading and secondary metabolic disorders from the glucose-based dialysate, and this could contribute to VaD. Moreover, our results showed that ESRD patients undergoing HD -- but not PD -- had a greater risk for AD. An imbalance between the production and clearance of β-Amyloid peptide (Aβ) in the central nervous system causes Aβ accumulation, and this may be the most important factor in the pathogenesis of AD [26]. A recent study found that serum Aβ levels were significantly higher in CKD patients who had never received dialysis than those who were receiving PD, and that ESRD patients undergoing PD and non-ESRD controls had comparable serum Aβ levels [27]. Other research indicated that HD only reduced serum Aβ levels by about 30% [28]. These findings suggest that the kidney has a role in the peripheral clearance of Aβ, and that PD might lower serum levels of Aβ. Future studies of AD in ESRD patients should consider alternative methods to increase the peripheral clearance of Aβ, and should further investigate this potentially important risk factor.

The strengths of this study are that it was a population-based, nationwide study that examined all validated cases of dementia which occurred in patients with ESRD over a 12-year period. All comorbidities and medical interventions were accurately recorded due to the national health insurance policy of Taiwan. However, the current study also has some limitations. First, we did not have measurements of inflammatory markers, direct measures of kidney function, nor measures of nutritional status. Although we adjusted for common health conditions in the multivariate analysis, there is a possibility that certain subclinical diseases may also have contributed to the observed cognitive decline in ESRD patients. Second, we relied exclusively on claims data, and this may have contributed to a bias in disease classification. In particular, doctors usually exclude reversible causes of dementia before making a diagnosis; however, because detailed hematological data were not available, we could not adjust for some potentially confounding factors for cognitive dysfunction in our population. Due to the limitations of the retrospective design of the current study, we are currently developing a detailed mechanism-based study of this topic by use of a prospective cohort design.

In conclusion, our study indicates that patients with ESRD who are undergoing HD or PD have a higher incidence of dementia than those without ESRD. Moreover, ESRD patients undergoing HD – but not PD – have a greater risk of AD than those without ESRD. Further research in this area is needed to identify the pathogenic mechanism(s) that connect ESRD with dementia, and to examine whether management of ESRD with PD rather than HD can effectively reduce the incidence of AD in these patients.

## MATERIALS AND METHODS

### Data collection

A universal National Health Insurance (NHI) programme was implemented in Taiwan during March 1995, and enrolled 96% of the total population at that time [29]. By the end of 1996, the Bureau of NHI (BNHI) had contracts with 97% of all Taiwanese hospitals and clinics to join the NHI system [30]. The BNHI accumulates all administrative and claims data for Taiwan, and the National Health Research Institute (NHRI) collaborated with the BNHI to establish an NHI research database. The NHRI safeguards the privacy and confidentiality of all beneficiaries, and provides health insurance data for research purposes only after obtaining ethical approval. To ensure the accuracy of the claims files, the BNHI conducts expert reviews on random samples for every 50–100 ambulatory and inpatient claims on a quarterly basis. False diagnoses can lead to imposition of severe penalties for the hospitals [31].

### Study population

We selected all patients from the NHI database who had ESRD and began maintenance HD or PD between 1 January 1999 and 31 December 2010, and who survived more than 90 days of renal replacement therapy (RRT) (*n* = 72,934). We excluded individuals younger than 18 years of age, because they had no appreciable risk of dementia. All HD and PD patients had catastrophic illness registration cards for treatment of ESRD (International Classification of Diseases, 9th revision, Clinical Modification [ICD-9-CM] code 585). In Taiwan, patients with ESRD can apply to the BNHI for catastrophic illness registration cards, so they have no copayments for long-term RRT. We excluded patients diagnosed with ESRD before the index date, those whose age or sex were not recorded, and those with any type of stroke (codes 430–438) before or within 90 days of the index date for ambulatory care (date of initiating dialysis). A total of 63,372 incident HD patients and 9,562 incident PD patients were identified during the study period. During the 12 months before the index date for ambulatory care, we obtained data on the following potential confounders (all of which are documented risk factors for dementia): hypertension (codes 401–405), diabetes (code 250), hyperlipidemia (code 272), CHD (codes 410–414 or 429.2), atrial fibrillation (AF) (code 427.31), and HF (codes 428).

Control subjects were randomly selected from the remaining subjects in the database using the same exclusion criteria, with exclusion of subjects with incident dementia prior to the index clinic visit. The control group consisted of randomly selected age- and sex-matched individuals who had no histories of ESRD or CKD. Participants with no CKD, but with documented hypertension, diabetes, hyperlipidaemia, CHD, AF, or HF, were included in the control group. The control-to-patient ratio was 1:1. We used propensity score matching to reduce selection bias resulting from nonrandom assignment. The age of each study subject was calculated as the time between the index date and the date of birth. Each patient was individually tracked from the index date for use of healthcare facilities to identify those who subsequently suffered from incident dementia (codes 290.0 to 290.4, 294.1, and 331.0 to 331.2), or until December 31, 2011.

### Statistical analysis

The primary endpoint was receipt of an ambulatory care visit or hospitalization for any type of dementia. The covariates were age, gender, region of residence (urban, suburban, or rural), and selected baseline comorbidities (hypertension, diabetes, hyperlipidemia, CHD, AF, and HF) [32–34]. Categorical variables are expressed as frequencies and percentages, and continuous variables as means and standard deviation (SD). We considered the above comorbidities only if the condition occurred in an inpatient setting, or if there were 2 or more ambulatory care claims recorded 1 year before or after the index ambulatory care visit. The baseline characteristics of the HD, PD, and control groups were compared using the SMD, calculated as the difference in means or proportions of a variable divided by a pooled estimate of the standard deviation of the variable [35]. This measure is not influenced by sample size, and is useful for comparison of cohorts in large observational studies. An SMD value of 0.1 or less indicates a negligible difference between groups [35].

Cox proportional hazard regression analysis was used to calculate HRs and 95% CI between ESRD (HD or PD) and dementia. The aHRs were obtained by correcting for demographic characteristics and comorbidities in multivariate Cox regression models. The dementia-free survival rates were estimated by the Kaplan-Meier method, and the log-rank test was used to calculate the significance of differences. We also examined the relationship between ESRD (HD or PD) and different dementia subtypes: AD (code 331.0), VaD (code 290.4), and UnD (codes 290.0 to 290.3, 294.1, and 331.1 to 331.2) [36]. All data analyses were conducted using SAS (ver. 9.3, SAS Institute, Cary, NC) for Windows, and the significance level was 0.05 in a two-sided test.

## Abbreviations

Aβ: β-Amyloid peptide
AD: Alzheimer's disease
AF: atrial fibrillation
aHR: adjusted hazard ratio
BNHI: Bureau of NHI
CHD: coronary heart disease
CKD: chronic kidney disease
ESRD: end stage renal disease
HD: hemodialysis
HF: heart failure
HR: hazard ratio
NHI: National Health Insurance
PD: peritoneal dialysis
SD: standard deviation
SMD: standardized mean difference
VaD: vascular dementia
UnD: unspecified dementia
